# Preparation of Zinc-Doped Hydroxyapatite Ceramics and Evaluation of Biocompatibility and Antibacterial Activity

**DOI:** 10.3390/jfb16030088

**Published:** 2025-03-04

**Authors:** José R. Guerra-López, Ana E. Bianchi, Marcelo A. Ramos, Mauro Ubertino, Verónica Ferraresi-Curotto, Jorge A. Güida, Katia Barbaro, Anna A. Zhukova, Victoria Yu. Grigorieva, Julietta V. Rau, Gustavo A. Echeverría

**Affiliations:** 1 Departamento de Ciencias Básicas, Universidad Nacional de Luján, Ruta 5 y 7, Luján 6700, Argentina; 2 Instituto de Ecología y Desarrollo Sustentable (INEDES, CONICET-UNLu), Luján 6700, Argentina; 3 Departamento de Física, Facultad de Ciencias Exactas, Universidad Nacional de La Plata and IFLP (CONICET, CCT La Plata), CC 67, La Plata 1900, Argentina; 4 Departamento de Ciencias Básicas Facultad de Ingeniería, Universidad Nacional de La Plata, 115 y 49, La Plata 1900, Argentina; 5 CEQUINOR (CCT-La Plata), Facultad de Ciencias Exactas, Universidad Nacional de La Plata, CC 962, La Plata 1900, Argentina; 6 Istituto Zooprofilattico Sperimentale Lazio e Toscana “M. Aleandri”, Via Appia Nuova 14111, 00178 Rome, Italy; 7 Department of Analytical, Physical and Colloid Chemistry, Institute of Pharmacy, I.M. Sechenov First Moscow State Medical University, Trubetskaya 8, Build. 2, 119048 Moscow, Russia; 8 Istituto di Struttura della Materia, Consiglio Nazionale delle Ricerche, ISM-CNR, Via del Fosso del Cavaliere 100, 00133 Rome, Italy

**Keywords:** zinc-doped hydroxyapatite, carbonate apatite, ceramics, antimicrobial properties, antimicrobial activity, mesenchymal stromal cells

## Abstract

Bacterial resistance and the demand for novel antibacterial strategies represent major challenges in contemporary medicine. In this study, zinc-doped hydroxyapatite (Zn-HA) samples with 3, 5, and 10 wt% Zn(II) were synthesized using wet precipitation synthesis and sintered at 700 and 800 °C. The samples were characterized by X-ray Diffraction, Fourier Transform Infrared Spectroscopy, Raman Spectroscopy, and Scanning Electron Microscopy. The antimicrobial properties of the Zn-HA were tested against four bacterial strains—*Staphylococcus aureus*, *Enterococcus faecalis*, *Salmonella typhimurium*, *Escherichia coli*—and the fungus Candida albicans. Both 5 wt% and 10 wt% Zn-HA effectively inhibited the growth of all microorganisms. Notably, 10wt% Zn-HA exhibited the best results, with inhibition rates of 50.2% against *S. aureus*, 36.5% against *E. faecalis*, 47.5% against *P. aeruginosa*, 31.8% against *E. coli*, and 24.7% against *C. albicans*. There were no significant differences in the growth of adipose mesenchymal stem cells between the prepared samples and the control. For osteogenic differentiation, dye uptake was 1.2 times higher for HA and 5 wt% Zn-HA, and 1.3 times higher for 3 wt% Zn-HA compared to the control. These results suggest that developed ceramics may be effective in regenerative medicine, paving the way for innovative treatments.

## 1. Introduction

Biomaterials are substances designed to replace or enhance the function of body components. Their compatibility with the body and their safety upon implantation are critical factors. Biomaterials can be either synthetic materials created in laboratories or natural materials derived from nature [1,2,3]. Among synthetic materials, numerous studies indicate that hydroxyapatite (HA, Ca_10_(PO_4_)_6_(OH)_2_) and tricalcium phosphate (β-TCP, Ca_3_(PO_4_)_2_) are in the list of the most promising candidates for biomedical implant applications [4,5,6]. However, there are several drawbacks to using these materials as long-term implants. Hydroxyapatite has limited mechanical properties and a slow osteointegration rate [1,2,4,5,6]. Additionally, it fails to prevent microbial growth over time. Ion exchange within the HA crystal structure involves the replacement of its constituent ions with other ions. This property led to research focused on developing substituted HAs [5,6,7,8,9,10].

A major limitation of HA is its slow rate of bone bonding. One approach to enhance bone fixation and reduce the risk of infection is the incorporation of natural source biological substances, bone morphogenetic proteins, antibiotics, etc. [3].

To address this issue, various apatites with different degrees of Ca(II) substitution by ions such as Ag(I), Na(I), Zn(II), Sr(II), and Mg(II) have been synthesized to enhance their osteointegration ability [5,6,7,8,9,10,11,12,13,14]. Furthermore, the interface between these materials and bone is a dynamic region where crystalline dissolution and re-precipitation take place. By precisely regulating their crystallinity, osteointegration can be enhanced.

Among the divalent metals, the replacement of Ca(II) with Zn(II) is particularly noteworthy, as both cations are abundant and play essential roles in biological systems [1,5,6,7,8,9]. Due to its superior in vivo biodegradation rate and enhanced biocompatibility for tissue regeneration, Zn(II) is considered an optimal candidate for biodegradable metallic materials compared to Fe(II) and Mg(II) ions [15].

Moreover, Zn(II) is particularly appealing due to its ability to stimulate osteoblastic activity in cells [1,5,6,7,8,9]. Zinc is involved in the activation of numerous enzymes related to metabolic processes, hormone regulation, and biomineralization. The presence of Zn influences the proliferative activity of osteoblast cells, promoting their growth. Additionally, Zn has been shown to inhibit osteoblastic bone resorption, further supporting its beneficial role in bone health. Consequently, Zn incorporation into biomaterials is highly advantageous for enhancing osteogenesis [1,9,13,14].

Recently, Zinc-doped hydroxyapatite (Zn-HA) has garnered attention for its bioactivity and antibacterial properties [9,10]. Studies on Zn-doped HA have provided valuable insights into its antibacterial activity, which could offer promising applications in the development of future biomaterials [11,13,14]. Research has consistently demonstrated that zinc-doped calcium phosphate materials can enhance bone repair while simultaneously exhibiting antibacterial properties [10,11,13,14]. However, most of these studies have focused on ceramics composed of a mixture of phosphates, mainly hydroxyapatite with tricalcium phosphate as a minor component. In these mixtures, zinc is primarily incorporated into tricalcium phosphate [13,14,16,17].

The formation of such phase mixtures during the synthesis or sintering process occurs due to limited substitution of calcium by zinc in the apatite structure. Zinc incorporation into the apatite structure leads to a reduction in bond distances, particularly between Zn-PO_4_^3−^ and Ca-Zn at the Ca_2_ site [5,6,18]. As a result, structural instability is induced, leading to a decrease in crystallinity and thermal stability [5,9,18]. Consequently, ceramics prepared at temperatures above 700 °C result in phase mixtures, such as Zn-β-TCP and HA, which restrict their medical applications [5,6,13,14].

We recently developed a new synthesis method in our laboratory that minimizes the incorporation of water into the apatite structure [18]. The synthesis was designed to produce Zn-HA with high crystallinity. For this purpose, NaOH was used instead of ammonium, and water content in the structure was limited through controlled evaporation. This process was optimized to continue during and after synthesis for more than 20 h, resulting in Zn-HA with high crystallinity and thermal stability exceeding 800 °C. In this context, the aim of the present study was to investigate the structural impact of Zn(II) doping on HA. Additionally, the antibacterial properties of the prepared ceramics are of particular importance for orthopedic applications, especially considering bacterial resistance issues and the need for new antibacterial strategies, which are key challenges in modern medicine.

In the present work, a series of different Zn-HA, with up to 10 wt% of Zn(II), were prepared by a wet precipitation method described in our previous work [18]. The prepared samples were sintered at two different temperatures, 700 and 800 °C. The sintered samples were characterized by X-ray diffraction (XRD), Fourier Transform Infrared Spectroscopy (FT-IR), FT-Raman Spectroscopy, and Scanning Electron Microscopy (SEM). The microbiology tests of the prepared zinc-doped HAs were carried out with Gram-positive bacteria (*Staphylococcus aureus* (*S. aureus*), *Enterococcus faecalis* (*E. faecalis*)), Gram-negative bacteria (*Salmonella typhimurium* (*S. typhimurium*), *Escherichia coli* (*E. coli*)), and *Candida albicans* (*C. albicans*) fungus. To evaluate toxicity and osteogenic differentiation, the growth and differentiation of adipose tissue-derived mesenchymal stem cells (AMSCs) were assessed using the MTT assay and Alizarin Red S staining.

## 2. Materials and Methods

In all synthesis procedures in this work, distilled water and analytical-grade reagents from Merck (Buenos Aires, Argentina) and Fluka (Buenos Aires, Argentina) were utilized.

### 2.1. Synthesis of Hydroxyapatite

HA was prepared following the method described by Gibson and Bonfield [19], which involves a reaction between Ca(OH)_2_ and H_3_PO_4_. At 98 °C, 0.3 M H_3_PO_4_ was added to a 0.2 M Ca(OH)_2_ solution at a rate of 0.30 mL/s to form a suspension. The resulting solid was then filtered, washed in distilled water, and dried overnight at 105 ± 1 °C.

### 2.2. Synthesis of Hydroxyapatite Doped with Zinc

As described in our previous work [18], the synthesis procedure was carried out in three steps. First, the reagents (Ca(OH)_2_, H_3_PO_4_, and ZnCl_2_) were calculated to achieve a (Ca^2^⁺ + Zn^2^⁺)/P molar ratio of 1.67. Next, NaOH was mixed in a stoichiometric proportion with ZnCl_2_. The mixture was then heated to 98 °C to evaporate water. The samples were prepared to achieve concentrations of 3, 5, and 10% zinc ions in mother solutions. All precipitation reactions were conducted at 98 °C, as with HA, but the pH was maintained at 9.5 by adding 0.1 M NaOH (instead of ammonium hydroxide solution). The obtained samples were labelled based on their Zn content in the mother solution (Zn/(Ca + Zn) × 100), expressed as mole fraction: HA, Zn-HA, Zn3-HA, Zn5-HA, and Zn10-HA.

Sintering was performed at 700 °C and 800 °C for 3 h.

### 2.3. Characterization

The composition of solid products was investigated by measuring the calcium, zinc, and phosphorus contents. Ca and Zn concentrations were quantified using atomic absorption spectroscopy (PerkinElmer Model 3110), while P levels were determined through spectrophotometric analysis. The solids obtained were sintered in an air atmosphere at two different temperatures, 700 and 800 °C, and were characterized by XRD, FTIR spectroscopy, FT-Raman spectroscopy, and SEM.

XRD patterns were collected using a PANalytical X’Pert Pro automatic diffractometer equipped with scintillation detector and graphite output monochromator utilizing CuKα radiation (λ = 1.5406 Å). Scans were performed in the range of 9° ≤ 2θ ≤ 100°, with a step size of 0.01° and a count time of 2 s.

For the IR spectra, the samples were prepared by grinding 1 mg of sample powder with 300 mg of KBr (infrared grade) in an agate mortar and then pelletizing under vacuum. The FTIR spectra were recorded at room temperature using a Shimadzu IR Prestige-21 Fourier transform spectrometer in the range of 4000–400 cm^−1^.

The Raman spectra of these compounds, recorded at room temperature in the range of 3500–100 cm^−1^, were acquired using a Bruker IFS-66 instrument equipped with a Raman FRA-106 accessory and an Nd:YAG laser operating in the NIR range (1064 nm).

For the morphological analysis, an FEI Quanta 200 ESEM Field Emission Scanning Electron Microscopy was used.

### 2.4. Toxicity, Osteogenic Differentiation, and Antimicrobial Tests

#### 2.4.1. Isolation of Mesenchymal Stromal Cells

Mesenchymal stromal cells were isolated from adipose tissue of a 24-month-old slaughtered male equine (AMSC) (Istituto Zooprofilattico Sperimentale del Lazio e Toscana (Rome, Italy)). The tissue was minced and placed into culture flasks with DMEM growth medium (Life Technologies, Paisley, UK) supplemented with 10% FBS (fetal bovine serum, Life Technologies, Paisley, UK) at 37 °C with 5% CO_2_.

#### 2.4.2. MTT Assay

To evaluate the toxicity of Zn-HA at different zinc concentrations (0%, 3%, 5%, and 10%), AMSCs were cultured in the presence of these substances, followed by the MTT assay [20], which measures the conversion of the yellow dye MTT (3-(4,5-dimethylthiazol-2-yl)-2,5-diphenyltetrazolium bromide) into purple formazan by mitochondria in living and viable cells. The amount of formazan was determined by measuring absorbance at 600 nm.

AMSCs at passage three were diluted to 40,000 cells/mL, placed into 24-well plates, and incubated at 37 °C with 5% CO_2_. After 24 h, the medium was replaced with a fresh growth medium containing 1 mg/mL of Zn-HA at different Zinc concentrations (0%, 3%, 5%, and 10%). All powders were sterilized in an autoclave at 121 °C for 25 min. The positive control consisted of AMSCs grown in the growth medium. Each experimental condition was tested 3 times. After 24 h of incubation, the medium was removed and replaced with the MTT solution (0.5 mg/mL, Sigma-Aldrich, St. Louis, MO, USA) in DMEM, followed by an additional 3-h incubation at 37 °C with 5% CO_2_. The MTT solution was then replaced with isopropanol (Sigma-Aldrich, St. Louis, MO, USA) for 30 min at room temperature. The optical density of the produced formazan was measured at 600 nm using a BioPhotometer (Eppendorf, Hamburg, Germany).

#### 2.4.3. Osteogenic Differentiation

AMSCs at the third passage were seeded (at 50,000 cells/mL) in 6-well plates and incubated at 37 °C and 5% CO_2_. After 24 h, an osteogenic differentiation medium consisting of growth medium supplemented with 50 µg/mL of ascorbic acid, 10 mM β-glycerophosphate, and 10^−7^ M dexamethasone (all from Merck, Darmstadt, Germany) was added. This medium contained 1 mg/mL of Zn-HA with varying Zn concentrations (0%, 3%, 5%, and 10%). The negative control consisted of AMSCs cultured in growth medium, while the positive control consisted of AMSCs cultured in osteogenic differentiation medium. Each experiment was replicated three times. AMSCs were maintained at 37 °C with 5% CO_2_, with specific medium refreshed every 48–72 h for a period of 21 days, and then stained with Alizarin Red S (Merck, Darmstadt, Germany), which highlights calcium deposits in red. AMSCs were fixed with 4% paraformaldehyde for 30 min at room temperature, rinsed, and then stained with 3% Alizarin Red S solution for 30 min. After four washes, red calcium deposits were visible in the extracellular matrix. Images were taken using an inverted optical microscope (Nikon, Eclipse TE 2000-U (AE Badhoevedorp, The Netherlands). In order to quantify the mineralization of the extracellular matrix, the stained AMSCs were treated with a 5% sodium dodecyl sulfate solution in 0.5 M HCl (Sigma-Aldrich, St. Louis, MO, USA) for 30 min. A 1 mL sample from each test was analyzed to get optical density at 490 nm using a biophotometer following the method described by Pang et al. [20].

#### 2.4.4. Antimicrobial Activity

The antimicrobial properties of Zn-HA powders at different zinc concentrations (0%, 3%, 5%, and 10%) were studied using four bacterial species (*E. coli* ATCC n. 25922, *S. aureus* ATCC n. 25923, *P. aeruginosa* ATCC n. 10145, and *E. faecalis* ATCC n. 29212) and one fungal species (*C. albicans* ATCC n. 10231). All powders were autoclaved at 121 °C for 20 min. All microorganisms were grown in Brain Heart Infusion (BHI) broth (DIFCO, Sparks, MD, USA) in the presence of 1 mg/mL of the various substrates for 24 h. Bacterial cultures were maintained at 37 °C, while the fungal culture was maintained at 28 °C. The positive control for each experiment consisted of growing each microorganism in BHI only. All experiments were performed in triplicate. Microbial proliferation was quantified by measuring the optical density at 600 nm using a BioPhotometer (Eppendorf, Hamburg, Germany).

#### 2.4.5. Statistical Analysis

We repeated the tests three times. The results of the MTT assay, Alizarin Red S quantification, and microbial growth rates were presented as mean ± standard deviation (SD) and analyzed statistically using Dunnett’s non-parametric test for multiple comparisons (using Sas JMP Statistical Discovery v14 Pro software, Milan, Italy). *p*-values ≤ 0.05 *, ≤0.01 **, and ≤0.001 *** were considered statistically significant.

## 3. Results

### 3.1. Chemical Analysis

Chemical analysis was carried out to control the synthesis procedure, and the obtained results are presented in Table 1. As expected, the obtained results are similar to those we reported previously [18]. In the Zn3-HA and Zn5-HA samples, the molar ratio values are close to the stoichiometric value of hydroxyapatite (1.67); however, in the Zn10-HA sample, the molar ratio is lower than expected. This could be attributed to a lower level of substitution of calcium for zinc in the apatite structure. As a consequence, it is reasonable to expect the formation of vacancies in the apatite lattice in Zn10-HA. Therefore, the thermal stability of Zn10-HA may be lower than that of the other Zn-HAs due to the presence of positively charged vacancies, which could be balanced by the loss of OH^−^ and/or PO_4_^3−^ ions [18,21,22,23].

### 3.2. Vibrational Spectra

The samples sintered at 700 and 800 °C were analyzed by Raman and IR spectroscopies to obtain information about the stability of apatite structure and possible local distortions induced by zinc.

#### 3.2.1. Infrared Spectra

**Figure 1:**
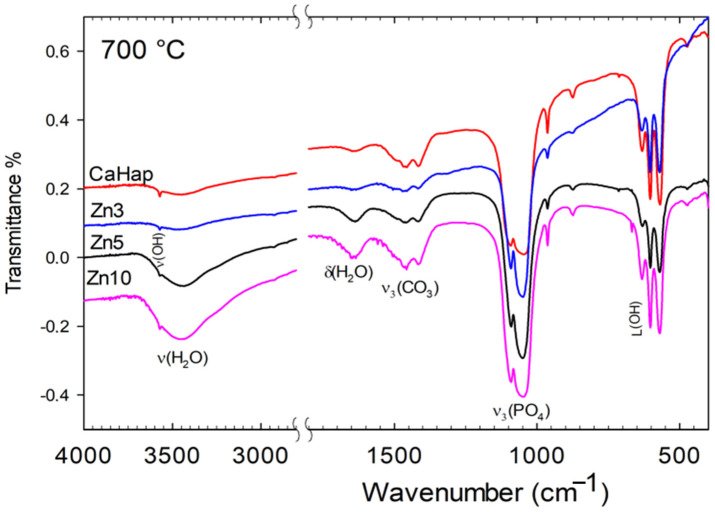
Infrared spectra of HA and Zn-HA sintered at 700 °C.

**Figure 2:**
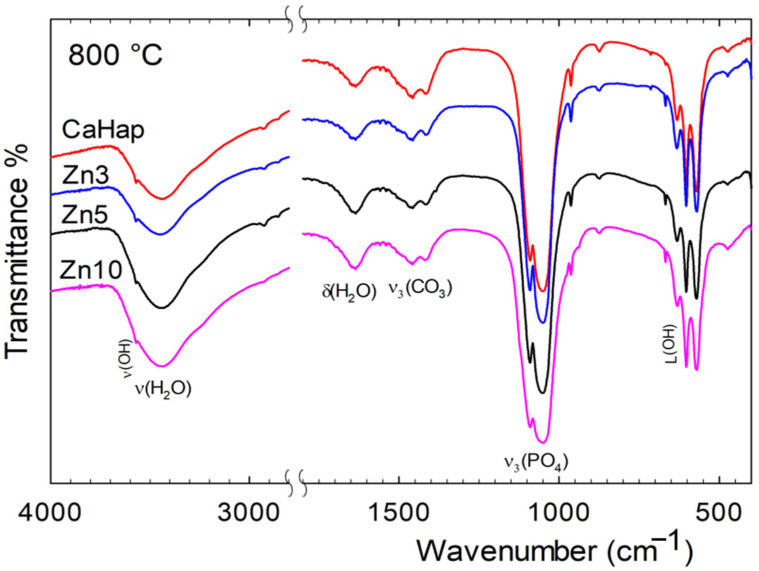
Infrared spectra of HA and Zn-HA sintered at 800 °C.

Figure 1 and Figure 2 show the IR spectra of HA and Zn-HA sintered at 700 and 800 °C. All the samples, treated at both temperatures, exhibit the characteristic bands of the CO_3_^2−^, PO_4_^3−^, and OH^−^ ions present in the carbonate apatite crystal lattice. In the case of samples treated at 700 °C, except Zn10-HA, there is no evidence of bands corresponding to the phases different from apatite. The main differences between the figures arise from slight shifts in band positions due to phosphate vibration modes, specifically from 1050 to 1047 cm^−1^ (ν_3_) and from 960 to 964 cm^−1^ (ν_1_). The shift effect was less pronounced for the bending modes at 602, 571 cm^−1^ (ν_4_), and 474 cm^−1^ (ν_2_). As previously reported [18,21], the stretching band of OH⁻ group vibrations undergoes a slight shift to lower wavenumbers, decreasing from 3572 cm^−1^ in HA to 3569 cm^−1^ in Zn-doped samples. In contrast, the corresponding librational mode follows the opposite trend, shifting to higher wavenumbers from 630 cm^−1^ to 633 cm^−1^ in HA at the same Zn concentration. The broad stretching bands at 1410 and 1470 cm^−1^, along with the weaker bands at 878 cm^−1^ and a shoulder at 870 cm^−1^ [5,24,25,26,27,28], clearly indicate the presence of carbonate groups in all the samples. The presence of a doublet at 870 cm^−1^ (ν_2_) suggests that CO_3_^2−^ occupies both the hydroxyl (type A) and phosphate (type B) positions in the structure [18,19,24,26,27,28]. The intensity of these bands indicates that CO_3_^2−^ preferentially occupies the phosphate site. The presence of carbonate ions in the samples results from the interaction between atmospheric carbon dioxide and a high concentration of OH^−^ ions present throughout the synthesis. It is noteworthy that the intensity of these bands does not increase with the increase in the amount of Zn(II).

Finally, for Zn10-HA, the weaker band at 690 cm^−1^ is attributed to the presence of ZnO in this sample [29,30,31]. The formation of ZnO can be explained by the following reactions:

Zn^2+^ + OH^−^ + CO_2_ ⟶ H_2_O + ZnCO_3_ (1)

ZnCO_3_ ⟶ ZnO + CO_2_ (2)

However, at higher temperatures, this behavior was observed in the spectra of all zinc-doped apatites, with a more intense band appearing at 690 cm^−1^, indicating increased carbonate decomposition. This observation is further supported by the decrease in all bands associated with carbonate ions across the entire spectral range.

Furthermore, at higher temperature, the spectra of all the samples, except for Zn10-HA, are very similar and exhibit the characteristic bands of CO_3_^2−^, PO_4_^3−^, and OH^−^ ions present in the carbonate apatite crystal, as described above. It is important to note that the presence of carbonate ion bands in the samples sintered at 800 °C further supports the idea that these ions are incorporated into the apatite structure.

On the other hand, in the case of Zn10-HA, new bands were observed in the 1200–900 cm^−1^ region. The bands at 972 and 945 cm^−1^, corresponding to ν_1_, and a shoulder at 1123 cm^−1^ (ν_3_) indicate the presence of the β-TCP phase, which arises as a result of the thermal process, as discussed in previous studies [7,18,21,32,33]. At lower frequency, the presence of the shoulder at 545 cm^−1^ due to bending of phosphate (ν_4_) confirms the presence of a mixture of phosphate phases. However, the intensity of the bands associated with β-TCP suggests that it is the minor component of the mixture.

Finally, it is important to note the absence of the 725 cm^−1^ band in the IR spectra of samples treated at 800 °C, which is typically associated with the pyrophosphate (P_2_O_7_^4−^) [18,21,32,33,34]. This absence indicates that there are no HPO_4_^2−^ ions in the solid phase. Therefore, the shoulder at 586 cm^−1^ in the spectra should be attributed to the presence of β-TCP in the solid phase.

#### 3.2.2. Raman Spectra

**Figure 3:**
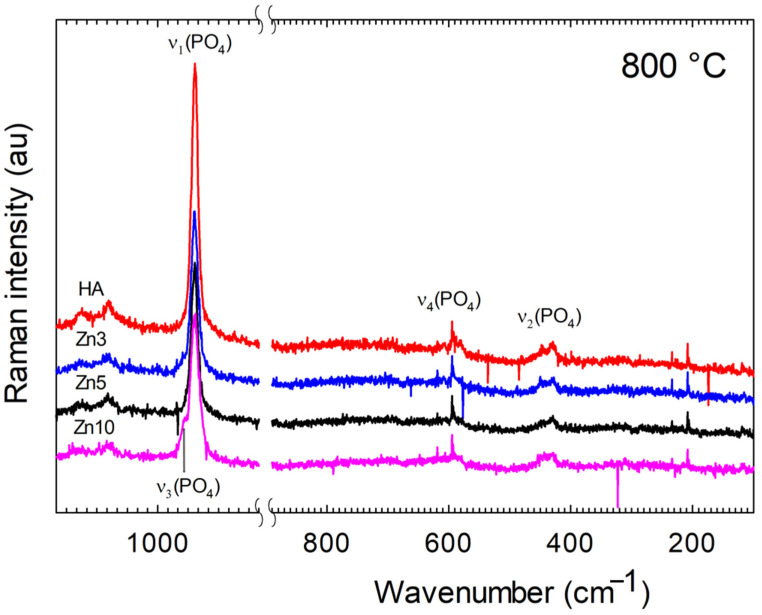
Raman spectra of HA and Zn-doped HA sintered at 800 °C (1100–400 cm^−1^ range).

Raman vibrational spectra of samples treated at 700 and 800 °C were obtained and analyzed. Due to the similarity observed with the results obtained by FTIR at 700 °C, only the spectra corresponding to samples sintered at 800 °C are presented in Figure 3. The bands corresponding to phosphate, carbonate, and hydroxyl ions are listed along with their internal modes in Table 2.

The Raman spectra of the prepared samples with different Zn amounts for selected spectral regions are shown in Figure 3; pure apatite spectrum is included for comparison. Due to the weak signal, the modes corresponding to carbonate ions provide limited information in the Raman spectra. Additionally, vibrational modes of hydroxyl ions were not observed in these spectra, despite the fact that these bands of weak intensity were reported in previous studies [21,35]. Therefore, our attention was focused on the phosphate modes. Table 2 presents a compilation of characteristic wavenumbers for the phosphate ion in hydroxyapatite. The intense band at 961 cm^−1^ is assigned to the ν_1_(PO_4_) stretching mode of the free tetrahedral phosphate ion. In contrast to the results reported by Nelson and Williamson [35], no splitting of this band was observed. In addition to the ν_1_(PO_4_) band, two ν_2_(PO_4_) bands, three ν_3_(PO_4_) bands, and two ν_4_(PO_4_) bands were resolved.

The comparison of HA to Zn3-HA and Zn5-HA shows few differences. The principal is due to a small shift from 961 to 963 cm^−1^ for the ν_1_(PO_4_) band when Zn is incorporated in the structure. No further shift could be detected with an increase in Zn content. The comparison between HA, Zn3-HA, and Zn5-HA reveals minimal differences, the most notable being a slight shift of the ν_1_(PO_4_) band from 961 to 963 cm^−1^ upon initial zinc incorporation into the structure. No further shifts were observed with increasing Zn(II) content. No bands corresponding to C–O vibrations were observed in the FT-Raman spectrum of HA or in the spectra of Zn-HAs. A thorough analysis revealed no other additional bands. In the case of Zn10-HA sintered at 800 °C, two new bands at 958 and 974 cm^−1^ assigned to ν_1_(PO_4_) evidence the partial decomposition of Zn-HA to β-TCP.

### 3.3. XRD Analysis

**Figure 4:**
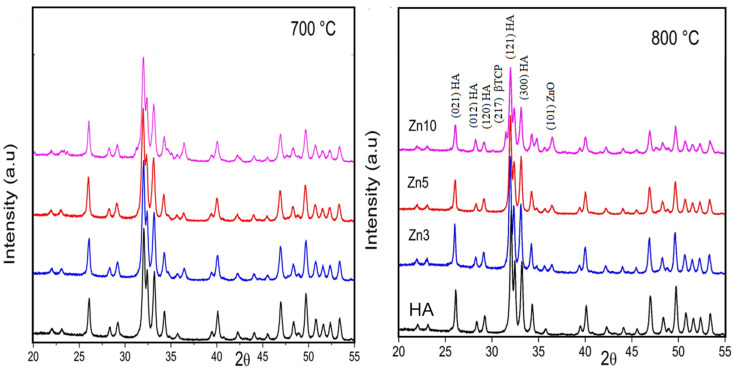
X-ray powder diffraction patterns of HA and Zn-HA samples in the range of 20°< 2θ < 55° sintered at 700 and 800 °C.

X-ray Diffraction patterns of the HA and Zn-HA samples sintered at 700 and 800 °C for 3 h are shown in Figure 4. The peaks identified at (002), (004), (034), (102), (131), (211), (213), (222), (300), (313), and (520) reflections in all the samples correspond to the hexagonal hydroxyapatite (JCPDS #9-432). However, in accordance with the results observed by FTIR and Raman, when treating them at 700 °C, two new peaks appear at 34.7 and 36.4 degrees. The last well-defined peak corresponds to a new phase identified as ZnO [30,36].

Notably, Zn3-HA and Zn5-HA exhibit similar thermal behavior. Upon heat treatment at 800 °C, the diffraction peaks become more intense and a new peak emerges at 35.1. This well-defined peak corresponds to the same phase observed in samples sintered at 700 °C.

For Zn10-HA, heat treatment at 700 °C results in three peaks in the XRD pattern at 34.6, 35.1, and 36.4 instead of the two observed in other samples. All these peaks correspond to ZnO, the same phase detected in thermally treated Zn3-HA and Zn5-HA. This finding suggests an increased decomposition of carbonate into CO_2_ and ZnO.

For the samples treated at 800 °C, the XRD patterns reveal an increased number of non-apatitic peaks at 30.1, 31.5, 34.5 (close to the 34.6 and 35.1 peaks observed at 700 °C), 37.8, 38.4, 41.6, and 47.6. These new peaks correspond to the β-(Ca,Zn)_3_(PO_4_)_2_ phase [7,18,21,31]. As previously discussed [18], the formation of this phase is attributed to the higher incorporation of Zn into the apatite structure, leading to vacancy formation and resulting in thermal instability.

### 3.4. SEM Images

**Figure 5:**
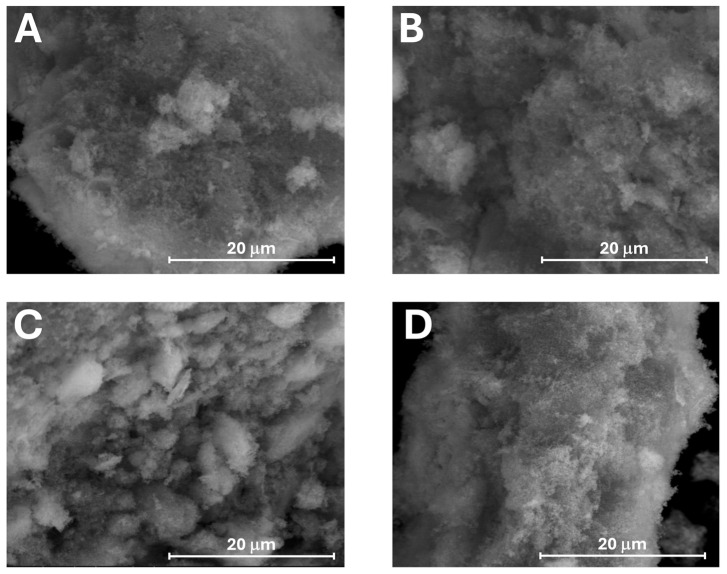
(**A**) HA, (**B**) Zn3-HA, (**C**) Zn5-HA, (**D**) Zn10-HA. Scale bar: 20 μm.

The morphologies of the synthesized HA and Zn-HA after sintering at 800 °C are presented in Figure 5.

**Figure 6:**
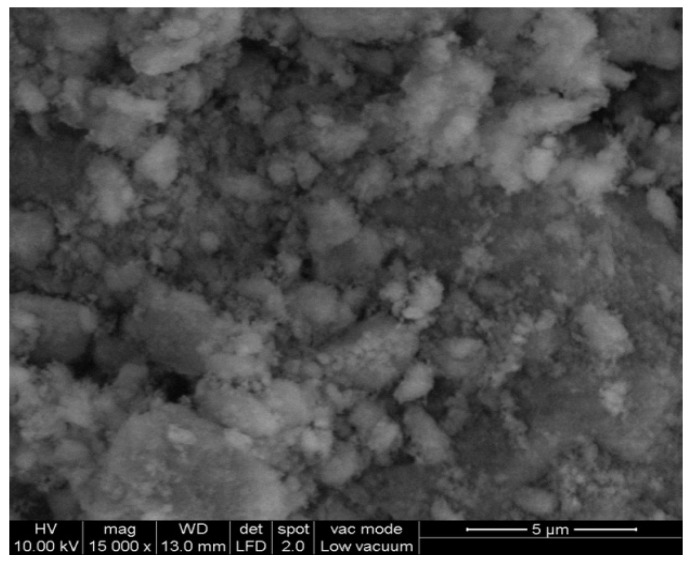
Cortical bone heated at 400 °C.

The SEM analysis revealed smaller grain sizes, which increased progressively from HA to Zn5-HA. The results indicated that the prepared HA nanocrystals initially consist of round particles that grow and acquire a more defined shape following Zn ion substitution in the apatite structure. This behavior may be attributed to the crystallinity of the powders. However, in the case of Zn10-HA, a reduction in grain size was observed, along with a morphology resembling that of pure HA. This phenomenon could be linked to the formation of a mixed-phase composition resulting from the decomposition of the apatite phase, as described above. Finally, it is important to note that all samples exhibit a dense morphology, closely resembling that of cortical bone carbonate apatite. This is further illustrated in Figure 6, which includes an image of bovine cortical bone treated at 400 °C.

### 3.5. MTT Assay Results

**Figure 7:**
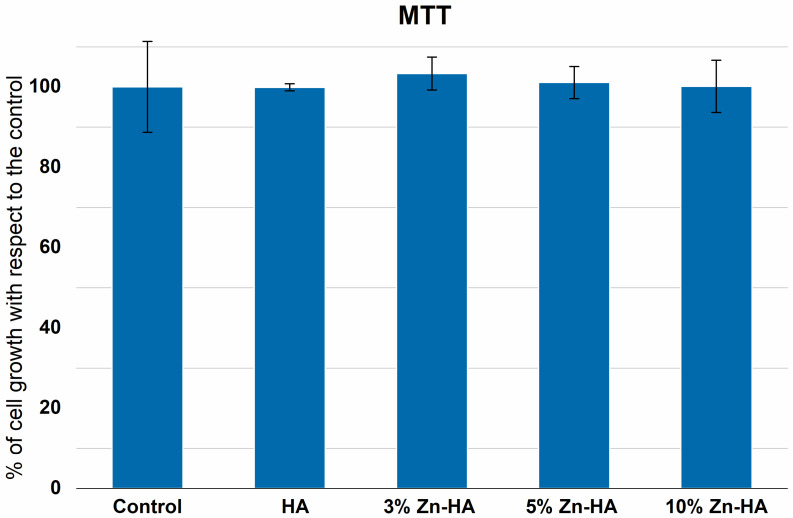
MTT assay: %growth of AMSCs cultured for 24 h with respect to the control in the absence (Cell control) and presence of Zn-HA at different Zn concentrations (0, 3, 5, and 10 wt%). The values were obtained from 3 experiments and are expressed as mean ± S.D. Control sample corresponds to 100% of the cell growth.

The MTT assay was used to evaluate the toxicity of the prepared Zn-HA samples at different Zn concentrations (0, 3, 5, and 10 wt%) on third-passage AMSCs. Figure 7 presents the percentage of cell growth obtained from three experimental trials. The cell control value corresponds to 100% growth of AMSCs without the prepared samples.

Specifically, the percentage of AMSC growth was 99.9 ± 0.9% in the presence of HA, 103.4 ± 4.1% in the presence of 3%Zn-HA, 101.1 ± 4.0% in the presence of 5%Zn-HA, and 100.1 ± 6.5% in the presence of 10%Zn10-HA, compared to AMSCs grown in the absence of the prepared samples. It can be concluded that the difference in %growth in all the experimental conditions, compared to the control, was not significant (Figure 7).

### 3.6. Osteogenic Differentiation Results

AMSCs were induced to differentiate into the osteogenic lineage both in the presence and absence of Zn-HAs at different Zn concentrations (0, 3, 5, and 10 wt%). Calcium deposits formed in the extracellular matrix, characteristic of osteogenic differentiation, were stained red using Alizarin Red S.

**Figure 8:**
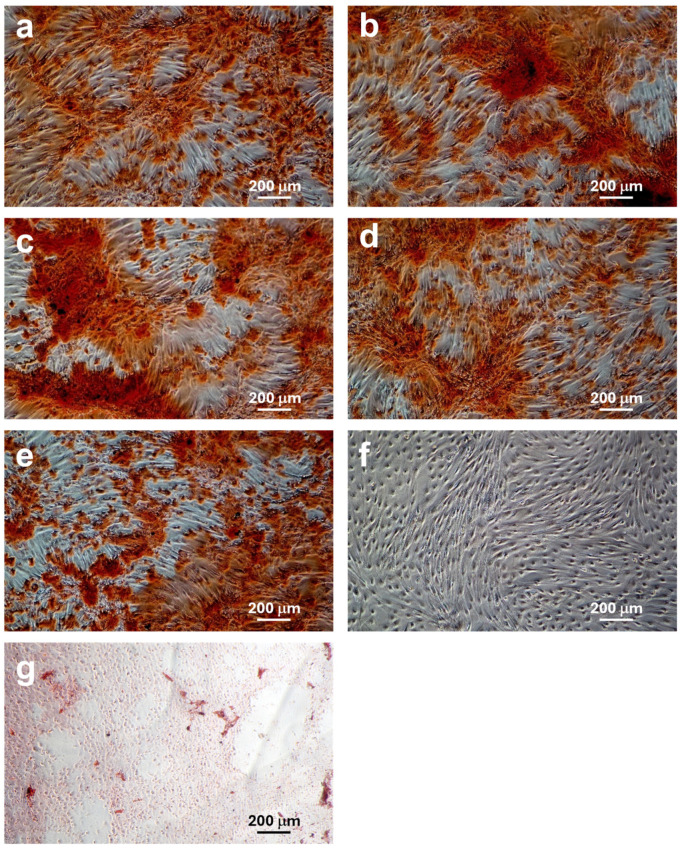
Alizarin Red S staining. AMSCs differentiated into the osteogenic lineage. (**a**) Positive control (AMSCs differentiated in the absence of samples). (**b**) AMSCs differentiated in the presence of HA. (**c**) AMSCs differentiated in the presence of Zn3-HA. (**d**) AMSCs differentiated in the presence of Zn5-HA. (**e**) AMSCs differentiated in the presence of Zn10-HA. (**f**) AMSCs grown in the growth medium. (**g**) AMSCs grown in the presence of HA, but without osteogenic differentiation medium. 10× magnification.

As shown in the images in Figure 8, AMSCs differentiate in the presence of all the prepared Zn-HAs at different Zn concentrations (0, 3, 5, and 10 wt%) into the osteogenic lineage. The images in Figure 8 depict the osteogenic differentiation of AMSCs under different experimental conditions after staining with Alizarin Red S.

**Figure 9:**
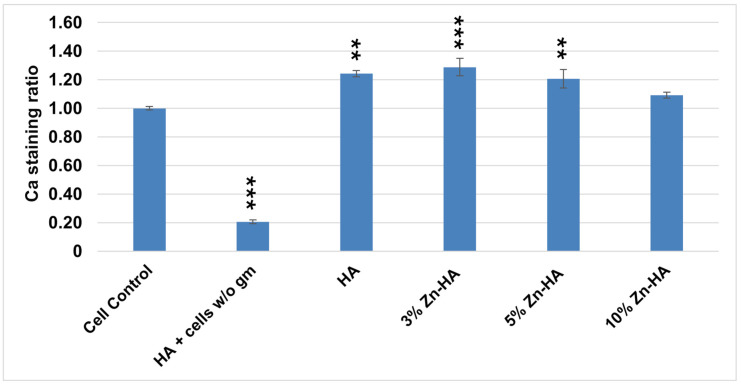
Calcium Alizarin Red staining ratio relative to the control on AMSCs differentiated into the osteogenic lineage in the absence (cell control) and presence of Zn-HAs at different Zn concentrations (0, 3, 5, and 10 wt%). HA + cells w/o gm test represents cells grown in the presence of HA, but without osteogenic differentiation medium. The reported values were obtained from 3 independent experiments and are expressed as mean ± S.D. Cell control corresponds to 1. *p*-values (Dunnett’s test) are *p* ≤ 0.01 ** and *p* ≤ 0.001 *** compared to the positive cell control.

The quantification of Alizarin Red S concentrations in the extracellular matrix of AMSCs is reported in Figure 9. The presence of the dye is proportional to the presence of calcium in the extracellular matrix following differentiation into the osteogenic lineage. Specifically, the presence of the dye was 1.24 times higher in the presence of HA, 1.29 times higher in the presence of Zn3-HA, 1.20 times higher in the presence of Zn5-HA, and 1.09 times higher in the presence of Zn10-HA (compared to the control). Calcium deposits stained with the dye were significant compared to the control for HA, Zn3-HA, and Zn5-HA. AMSCs grown in the presence of HA and without growth medium (HA + cells w/o gm) produced calcium deposits highlighted with 0.21 compared to the cell control.

### 3.7. Antimicrobial Activity Results

**Figure 10:**
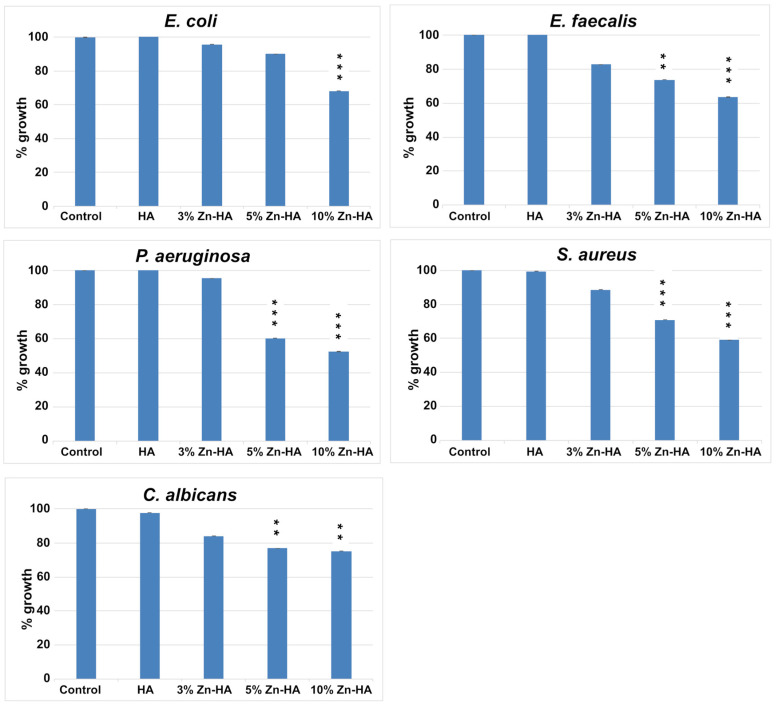
Growth % of *E. coli*, *E. faecalis*, *S. aureus*, and *P. aeruginosa* bacterial strains and *C. albicans* fungus grown in the presence and absence of Zn-HA with different Zn concentrations (0, 3, 5, and 10 wt%). The reported values represent the average value obtained from 3 experiments ± S.D. compared to the positive control (100%). *p*-values (Dunnett’s test): ≤0.01 ** and ≤0.001 ***.

The growth of microorganisms (*E. coli*, *S. aureus*, *P. aeruginosa*, *E. faecalis*, and *C. albicans*) in the absence and presence of Zn-HAs at different Zn concentrations (0, 3, 5, and 10 wt%) is shown in Figure 4. The positive control for each experiment consisted of each microorganism grown in the absence of substrates for 24 h at optimal growth temperature. The growth percentage and standard deviation (SD) obtained as an average of 3 independent experiments is presented in Figure 10.

The growth of *E. coli* was 100.40% in the presence of HA, 95.69% in the presence of 3% Zn-HA, 90.05% in the presence of 5% Zn-HA, and 68.20% in the presence of 10% Zn-HA compared to the control. Only the growth inhibition in the presence of 10% Zn-HA was statistically significant compared to the control.

The growth of *E. faecalis* was 101.62% in the presence of HA, 82.73% in the presence of 3% Zn-HA, 73.58% in the presence of 5% Zn-HA, and 63.54% in the presence of 10% Zn-HA compared to the control. Only the growth inhibitions in the presence of 5% Zn-HA and 10% Zn-HA were statistically significant compared to the control.

For *P. aeruginosa*, the growth was 100.26% in the presence of HA, 95.30% in the presence of 3% Zn-HA, 60.24% in the presence of 5% Zn-HA, and 52.51% in the presence of 10% Zn-HA compared to the control. Only the growth inhibitions in the presence of 5% Zn-HA and 10% Zn-HA were statistically significant compared to the control.

*S. aureus* grew 99.45% in the presence of HA, 88.66% in the presence of 3% Zn-HA, 70.96% in the presence of 5% Zn-HA, and 59.05% in the presence of 10% Zn-HA compared to the control. Only the growth inhibitions in the presence of 5% Zn-HA and 10% Zn-HA were statistically significant compared to the control.

For *C. albicans*, the growth was 97.61% in the presence of HA, 84.11% in the presence of 3% Zn-HA, 76.98% in the presence of 5% Zn-HA, and 75.26% in the presence of 10% Zn-HA compared to the control. Only the growth inhibitions in the presence of 5% Zn-HA and 10% Zn-HA were statistically significant compared to the control.

## 4. Discussion

The XRD, FTIR, and FT-Raman analyses revealed the presence of carbonate ions in both HA and Zn-doped HA samples sintered at 700 °C and 800 °C. Absence of peaks corresponding to carbonate compounds, ZnCO_3_ or CaCO_3_, suggests that these ions are incorporated into the apatite structure.

These results are consistent with those of the FTIR and FT-Raman analyses, which showed that the band positions are indicative of this ion occupying both the hydroxyl (type A) and phosphate (type B) positions [18,19,24,25,26,27,28]. Furthermore, the absence of the band at 725 cm^−1^, attributed to the formation of the pyrophosphate phase (P_2_O_7_^4−^) as a result of the thermal decomposition of HPO_4_^2−^ at temperatures above 700 °C, provides evidence for the absence of this ion in the apatite structure. The positions of carbonate bands are another important aspect to highlight, as the presence of CO_3_^2−^ ions contributes to the formation of ZnO during the sintering process. The incorporation of carbonate into the HA structure can be justified by the high concentration of OH^−^ ions during synthesis and the interaction with atmospheric CO_2_ according to reaction (3):

Ca^2+^ + 2OH^−^ + CO_2_ ⟶ H_2_O + CaCO_3_ (3)

The presence of these ions in the structure of apatite is related to the compensation of charge in the structure due to reaction (4):

Ca^2+^ + PO_4_^3−^ + OH^−^ ⟶ V_Ca_ + CO_3_^2−^ + V_OH_ (4)

where V represents vacancy in the structure. The incorporation of carbonate ions results in instability that is additionally generated by Zn ions present in the structure [7,26,35]. However, there is some evidence suggesting that the incorporation of carbonate into the solid is lower. The intensity of the bands associated with carbonate-apatite in the spectra, as well as the lines corresponding to ZnO in the XRD pattern, were observed in the Zn3-HA and Zn5-HA samples. Additionally, the chemical analysis (see Table 1) and the reduced incorporation of water, as determined in our previous work [18], further support this idea.

However, future studies will require Rietveld refinement analysis to confirm the incorporation of carbonate ions into the hexagonal phase at the phosphate and hydroxyl positions. This was not the focus of the current study.

On the other hand, it is important to note that ZnO formation begins at 700 °C for Zn10-HA, whereas for the other zinc-doped HA samples, it occurs at 800 °C. Furthermore, in the case of Zn10-HA sintered at 800 °C, the partial decomposition of the apatite structure should be taken into account. As mentioned in [18], this phenomenon may be attributed to the partial substitution of calcium by zinc, leading to the formation of vacancies. This process is represented by reaction (5):

(Ca,Zn)_10−x_(PO_4_)_6_(OH)_2−2x_ ⟶ z β(Ca,Zn)_3_(PO_4_)_2_ + (Ca,Zn)_10−x−3z_(PO_4_)_6−2z_(OH)_2−2x−y_O_y_V_y_ + y H_2_O (5)

where z is the molar fraction of the β-TCP phase. As the temperature increases, the decomposition of Zn-HA facilitates the formation of the β-TCP and ZnO. ZnO is studied for its antibacterial properties in both micro- and nanoscale formulations [37,38,39,40,41,42,43,44]. Therefore, its presence in the solid is not expected to compromise its potential as a biomaterial.

Another aspect of Zn incorporation in HA is its impact on biocompatibility and antibacterial properties. Our investigation of Zn-HAs with various Zn(II) concentration (0, 3, 5, and 10 wt%) highlights their significance in tissue engineering and regenerative medicine. The MTT assay and AMSC osteogenic differentiation test confirmed that the developed materials are non-toxic and support AMSC growth at levels comparable to the control.

Another important aspect analyzed in this work is the ability of Zn-HAs to inhibit the growth of bacteria such as *E. coli*, *S. aureus*, *P. aeruginosa*, *E. faecalis*, and the fungus *C. albicans*. Obtained results demonstrated their potential to prevent infections, enhancing their suitability for clinical applications. Notably, 10% Zn-HA effectively inhibited the growth of all tested microorganisms.

The results presented above indicate that after heat treatment at 700 °C, a dense Zn-doped apatite can be obtained with Zn(II) concentrations of 3, 5, and 10 wt%, along with lower carbonate incorporation into the structure. Furthermore, spectroscopic and crystallographic analyses (see Figure 1, Figure 2, Figure 3 and Figure 4) confirm that increasing the temperature promotes the formation of the β-TCP phase. Both β-TCP and HA demonstrated their potential as biomaterials, with the main difference being the rate of osteointegration [1,2,4,15].

Thus, the sintering process at 700 °C and 800 °C appears to be optimal for producing two distinct bioceramics. Notably, a low amount of ZnO is formed due to the decomposition of carbonate. However, this does not negatively impact the properties of the developed materials.

## 5. Conclusions

In the present work, we synthesized dense Zn-HAs with varying Zn substitution wt% (3, 5, and 10 wt%) by means of the wet precipitation method. FTIR and XRD analyses revealed that a carbonate apatite A-B co-substituted with Zn(II) was obtained. The results showed that the thermal stability of the carbonate apatite phase remained intact up to 700 °C for all the sintered samples. However, at 800 °C, the Zn10-HA sample exhibited a phase mixture primarily consisting of carbonate apatite, with smaller concentrations of β-TCP and ZnO.

The antimicrobial activity test demonstrated that all Zn-HA samples synthesized in this study inhibited the growth of all five tested microorganisms. However, the best results were obtained for 10 wt% Zn-HA, with an inhibition percentage of 50.2% against *S. aureus*, 36.5% against *E. faecalis*, 47.5% against *P. aeruginosa*, 31.8% against *E. coli*, and 24.7% against *C. albicans* compared to the control.

The AMSC growth percentages were 99.9% for HA, 103.4% for 3 wt% Zn-HA, 101.1% for 5 wt% Zn-HA, and 100.1% for 10 wt% Zn-HA compared to the control group. No statistically significant differences in cell growth were observed under any of the experimental conditions. Regarding osteogenic differentiation, the dye uptake was 1.24 times higher for HA, 1.29 times higher for 3% Zn-HA, and 1.20 times higher for 5% Zn-HA, showing significant increases compared to the control. This difference was not significant for 10% Zn-HA.

Our findings prove the development of two types of ceramics with promising clinical potential, given their significant antibacterial activity, improved structural properties, and enhanced cell viability. One type is composed of a mixture of Zn-HA and β-(Ca,Zn)_3_(PO₄)_2_ (Zn10-HA), while the other consists solely of Zn-HA (Zn5-HA). The developed materials support mesenchymal stem cell growth and differentiation without exhibiting toxic effects. Additionally, their ability to inhibit the growth of microorganisms establishes them as promising tools in regenerative medicine, paving the way for innovative and effective treatments. The synergistic combination of biocompatibility and antibacterial properties in the Zn-HA materials underscores their potential as essential components for the future of tissue engineering.

## Figures and Tables

**Table 1:** Atomic absorption spectroscopy analysis of pure and Zn-HA samples.

Sample	Calcium ^a^	Phosphorous ^b^	Zinc ^a^	Molar Ratio (Ca^2+^ + Zn^2+^)/P
HA	0.96 ± 4	0.576 ± 3	----------	----------
Zn3-HA	0.93 ± 1	0.558 ± 3	0.004 ± 2	1.67
Zn5-HA	0.91 ± 1	0.555 ± 3	0.006 ± 2	1.65
Zn10-HA	0.88 ± 3	0.588 ± 4	0.009 ± 2	1.51

^a^ The chemical analysis values are presented in at%. ^b^ Phosphorus content was measured spectrophotometrically.

**Table 2:** Raman band positions for HA and Zn-HA at 700 °C.

Modes	HA	Zn3-HA	Zn5-HA	Zn10-HA
PO_4_^3−^ (ν1)	961	963	964	964
PO_4_^3−^ (ν3)	1075, 1048, 1029	1075, 1048	1075, 1048	1048, 1029
PO_4_^3−^ (ν4)	608, 593, 584	593, 584	592, 582	593, 584
PO_4_^3−^ (ν2)	448, 432	432	432	432
CO_3_^2−^ ions	Absent	Absent	Absent	Absent
OH^−^ ions	Absent	Absent	Absent	Absent

## Data Availability

The experimental data are available upon a reasonable and official request to the corresponding authors.
